# Prefrontal activity during the emotional go/no-go task and computational markers of risk-based decision-making predict future relapse in alcohol use disorder

**DOI:** 10.3389/fpsyt.2022.1048152

**Published:** 2023-01-04

**Authors:** Jun Sasaki, Toshio Matsubara, Chong Chen, Yuko Fujii, Yoko Fujita, Masako Nakamuta, Kumiko Nitta, Kazuteru Egashira, Takashi Hashimoto, Shin Nakagawa

**Affiliations:** ^1^Division of Neuropsychiatry, Department of Neuroscience, Yamaguchi University Graduate School of Medicine, Ube, Japan; ^2^Koryo Hospital, Ube, Japan; ^3^Egashira Clinic, Kitakyushu, Japan

**Keywords:** alcohol use disorder, decision-making task, emotional go/no-go task, functional near-infrared spectroscopy (fNIRS), relapse, predictors

## Abstract

**Aim:**

To longitudinally examine if the results of cognitive tasks or brain function during emotional or cognitive tasks can predict relapse in alcohol use disorder.

**Methods:**

We selected 41 patients with alcohol use disorder during hospitalization. Functional near-infrared spectroscopy (fNIRS) measured the relative change in oxygenated hemoglobin in the frontotemporal areas during an emotional go/no-go task and verbal fluency task (VFT). They performed the N-back and risk-based decision-making tasks for determining working memory or risk-based decision-making. The presence of relapse 6 months following discharge was the primary outcome.

**Results:**

Twenty-four patients (21 men, three women) remained abstinent, whereas 17 (14 men, three women) relapsed. Compared with the abstinent group, those with relapse displayed significantly decreased activation in the right frontotemporal region during the emotional go/no-go task, significantly shorter reaction time to non-emotional stimuli, and greater risk preference in the risk-based decision-making task. In the abstinent group, we observed a negative correlation between oxygenated hemoglobin and the craving scale. A logistic regression analysis demonstrated that the risk of relapse increased with smaller oxygenated hemoglobin in the right frontotemporal region (odds ratio = 0.161, *p* = 0.013) and with greater gambling thoughts (odds ratio = 7.04, *p* = 0.033).

**Conclusion:**

Decreased activation in the right frontotemporal region in response to an emotional stimulus and risk preference could predict relapse in alcohol use disorder.

## Introduction

In 2016, the harmful use of alcohol caused approximately 3 million deaths (5.3% of all deaths) and 132.6 million disability-adjusted life years worldwide, and the mortality from alcohol consumption was higher than that from tuberculosis (2.3%), human immunodeficiency virus infection/acquired immune deficiency syndrome (1.8%), diabetes (2.8%), and hypertension (1). Alcohol use disorders (AUD) require early treatment because of the extensive impairment of daily functioning due to physical disabilities, such as liver damage and pancreatitis caused by drinking, and behavioral symptoms, including withdrawal, tolerance, and craving. However, the high relapse rate of AUD is challenging in the treatment of alcohol-related disorders. Neto et al. demonstrated that 39.2% of the patients with AUD remained abstinent 6 months following discharge (2). In addition, during the year after treatment, about 25% of the patients with AUD remained continuously abstinent, thus suggesting the difficulty of continuing abstinence (3). These findings suggest the difficulty of continuing abstinence. Biological markers are required to predict future relapse following discharge, identify high-risk inpatients, and provide effective interventions.

Cognitive dysfunction is one of the leading characteristics on psychiatric disorders including AUD. Actually, patients with AUD report impaired impulse control (4, 5), executive dysfunction (6, 7) and impaired risk-based decision-making (8), compared with controls. Moreover, the group that relapsed after 3 months displayed greater impulsivity than the group that remained abstinent (9). A recent review demonstrated a correlation between impulsivity and relapse in AUD (10). Noël et al. reported on a significantly stronger impairment of working memory in the drinking group than that in the abstinent group (11). Previous reports using the Iowa Gambling Task (IGT, (12, 13), which measures risk-based decision-making, demonstrated that lower performance on the test at the beginning of observation was positively correlated with alcohol consumption in a 2-year prospective study of low drinkers (14), whereas a 6-month prospective study did not observe a difference in the Iowa Gambling Test performance between the relapse and abstinent groups (15). The reason of this conflicting results with IGT may be due to the fact that IGT involves multiple cognitive processes and poor performance on the IGT can result from greater risk seeking, lower loss aversion, or compromised reinforcement learning of the reward/loss contingencies. That is, the task does not allow reliable evaluation of each cognitive process and to predict the prognosis of AUD, more precise economic theory-based tasks are required.

Despite these results suggesting that the mentioned impairments may be biological predictors of relapse in patients with AUD, they seldom included longitudinal studies. Therefore, we aimed to examine if these three factors can be used to predict the prognosis of patients with AUD 6 months following discharge from the hospital. Importantly, a recent meta-analysis of fMRI showed that patients with AUD demonstrated hyperactivation of the prefrontal cortex to alcohol cues compared to controls (16), suggesting impaired top-down emotional regulation. Thus, we intended to use functional near-infrared spectroscopy (fNIRS) to measure brain activation in the frontotemporal regions during the emotional go/no-go task and verbal fluency task (VFT) to assess neurocognitive foundations during cognitive and emotional regulation tasks. The emotional go/no-go task, which requires participants to discriminate stimuli of different emotional valence as well as to inhibit a prepotent response, measures inhibitory control that is supported by the prefrontal cortex. VFT requires participants to generate as many as possible words according to certain rules and has been shown to involve executive functions and the prefrontal cortex. Previous studies have reported poor performance of patients with AUD in the emotional go/no-go task, indicating impaired impulse control (4, 5). Also, patients with AUD also produce less words in the VFT, indicating compromised executive function (7). Based on these evidence, we hypothesized that patients with relapse would display compromised impulse control (specifically, shorter reaction time), accompanied by decreased brain activation response to emotional stimuli during the emotional go/no-go task or to VFT.

Furthermore, to evaluate working memory performance, we used the commonly employed n-back task and hypothesized that patients with AUD would demonstrate decreased working memory. For the evaluation of risk-based decision-making, rather than IGT, we used a risk-based decision-making task that allow economic theory-based computational modeling of the underlying cognitive process. Specifically, following our previous study (17), we dissected risk preference into two parameters, utility sensitivity and probability weighting that allows more precise evaluation of risk seeking behaviors. Utility sensitivity represents general risk preference while probability weighting focuses on potentially different risk preference at small vs. large probabilities. Based on previous reports indicating that greater risk seeking may be associated with AUD, we hypothesized that patients with relapse would show altered utility sensitivity and probability weighting that indicate enhanced risk seeking.

## Materials and methods

### Design

**FIGURE 1:**
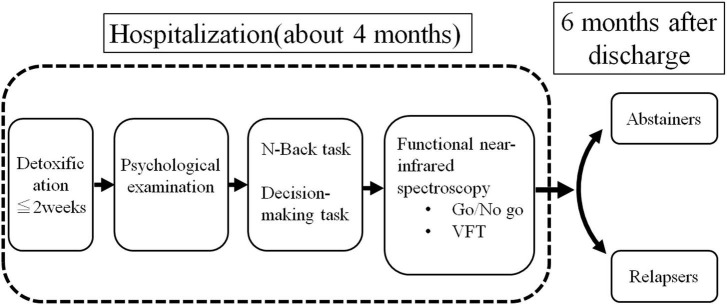
Study design.

This longitudinal study was conducted at the Koryo Hospital and Yamaguchi University Hospital. Figure 1 illustrates the flow of the study. Following admission, the patients underwent detoxification treatment. The detoxification period was 1 week. We administered infusions once daily for 7 days, diazepam 15 mg 3 days and diazepam 7.5 mg 4 days for a total of 7 days. After detoxification, psychological tests including self-reports was performed. Subsequently, we performed the N-back task and risk-based decision-making task on the participants using a computer. Eventually, we measured their brain activity using fNIRS with the emotional go/no-go task and VFT during hospitalization. We performed the task in the first month after admission and the fNIRS measurements in the first month and a half. Relapse, the outcome of this study, was defined as self-reported or reported by a relative or close friend at the Koryo Hospital outpatient clinic 6 months following discharge, with ≥60 and ≥40 g alcohol consumption for men and women, respectively, during the follow-up (6 months) according to Beck et al. (18).

### Participants

All participants were recruited from the patients admitted to the Koryo Hospital, which specializes in the treatment of AUD. This study was approved by the institutional review boards of the Koryo Hospital and Yamaguchi University Hospital. It was conducted in accordance with the ethical standards of the Declaration of Helsinki. The age of the participants ranged from 20 to 70 years, and all met the Diagnostic and Statistical Manual of Mental Disorders, 5th Edition (19) for alcohol dependence. Written informed consent was obtained from all participants. The exclusion criteria were as follows: (1) a history of the loss of consciousness for more than 1 h owing to severe head injury or brain tumor; (2) a history of or current treatment for a neurodegenerative disease; (3) alcohol withdrawal syndrome with impaired consciousness; and (4) dementia or suspected dementia with a score ≥24 on the Mini-Mental State Examination (20, 21) of cognitive function. As a standard of care, all participants participated in a treatment program based on 12 STEP meetings during hospitalization. During the follow-up after discharge, the participants were encouraged, but not required, to participate in the hospital’s standard treatment program, including outpatient visits and participation in day care. Moreover, they were encouraged to join self-help groups. Following enrollment, they completed an interview to assess alcohol dependence and comorbid psychiatric disorders. Furthermore, we performed interviews to determine the average number of drinking days per week and the average amount of alcohol consumed per day during the year prior to hospitalization. We assessed the severity of AUD prior to admission using the Alcohol Use Disorders Identification Test (AUDIT) (22), vulnerability to alcohol, or craving using the Stimulus-Induced Vulnerability subscale of the Alcohol Relapse Risk Scale (ARRS-SV) (23). We assessed depression with the Beck Depression Inventory-II (BDI-II) (24) and Structured Interview Guide for the Hamilton Depression Rating Scale-17 (25). Moreover, impulsivity was assessed with the Barratt Impulsiveness Scale 11th (BIS-11) (26). The dominant arm was evaluated using the Edinburgh Handedness Inventory (27).

### fNIRS instrument

**FIGURE 2:**
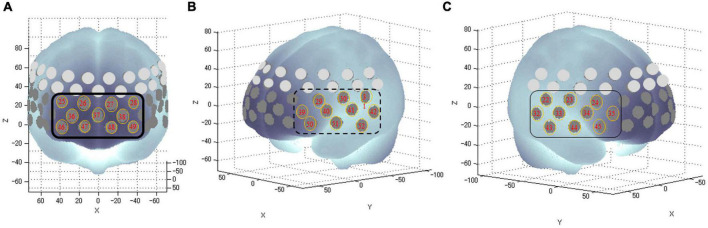
Anatomical areas of the brain measured by fNIRS. The numbers in tangerine-colored circles represent the channels of measurement in the anatomical area **(A)**: The frontopolar area (channel #25–28, 36–38, and 46–49) corresponding to the superior and middle frontal gyri; **(B)**: Left frontotemporal areas (channel #29–31, 39–42, and 50–52) corresponding to the inferior and middle frontal gyri; and **(C)**: Right frontotemporal areas (channel #22–24, 32–35, and 43–45) corresponding to the anterior portion of the superior and middle temporal gyri.

Functional near-infrared spectroscopy is a device that uses near-infrared light to measure blood dynamics on the surface of the prefrontal cortex in real time. It offers the advantages of no radiation exposure, lower cost, smaller size than fMRI, and the ability to measure while sitting (28, 29). We used a continuous-wave fNIRS system (ETG-4,000; Hitachi Medical Corporation, Tokyo, Japan) to measure brain function. Relative changes in oxygenated hemoglobin concentrations (oxy-Hb) were monitored. The time resolution was 0.1 s. We used multichannel probe holders (3 × 11), each consisting of 17 eliminating and 16 detecting probes alternately arranged at an inter-probe distance of 3 cm, thus resulting in 52 channels per set. The channels were placed in accordance with the international 10–20 system. The lowest probes were positioned along the Fp1–Fp2 line. We corrected for motion artifacts using the moving average method according to a previous study (30), which removed short-term motion artifacts from the analyzed data to smooth out these concentration change (moving average windows: 5 s), and the algorithm method to exclude channels contaminated with rhythmic signals that indicated noise and motion artifacts. We measured physiological noise in this study, such as heart rate. There was no significant difference in heart rate between the two groups (*p* = 0.30). In addition, channels with remarkable motion artifacts were deleted following blinded assessment by the first author (JS) and a co-author (TM). Data were analyzed using the integral mode, in which the pre-task baseline during the control block was determined as the mean (oxy-Hb) of the last 10 s in the post-task period. The data between the two baselines were fitted linearly. Based on previous studies (28, 29), we measured the frontal and temporal areas using 31 channels (channels #22–52). We anatomically identified the areas using a virtual registration method with an automated anatomical labeling that enabled the registration of fNIRS channel positions in the standard brain space (31). These channels were classified into the following three areas according to previous fNIRS studies: the frontopolar area (channels #25–28, 36–38, and 46–49, corresponding to the superior and middle frontal gyri), left frontotemporal area (#29–31, 39–42, and 50–52, corresponding to the anterior portion of the superior and middle temporal gyri), and right frontotemporal area (#22–24, 32–35, and 43–45, corresponding to the inferior and middle frontal gyri) (Figure 2). For analyzing the fNIRS data, we used (oxy-Hb) during the task as an outcome measure for statistical analyses because it reflected the activation of gray matter in the brain. We used the integral value of (oxy-Hb), the change in (oxy-Hb) over the period of the targeted task, for the analysis (28, 29, 32–34).

### Stimuli and experimental procedure

**FIGURE 3:**
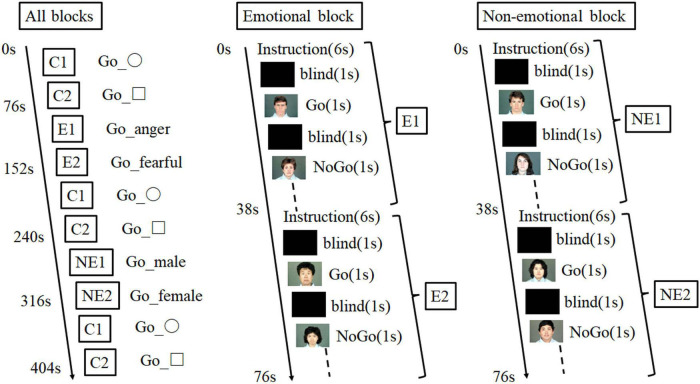
Experimental design of the emotional go/no-go task.

According to our previous study (34), the emotional go/no-go task consisted of five blocks as follows: one emotional block with emotional faces of anger or fear, one non-emotional block with neutral faces, and three control blocks with geometric shapes (Figure 3). There were 32 trials in each run. We selected facial photographs from the Japanese and Caucasian Facial Expressions of Emotion and Neutral Faces (35). We used presentation soft (Neurobehavioral System, Inc) to show the photographs. For the go trials, the participants responded by rapidly pressing a button on a keypad with the index finger of their preferred hand upon the appearance of a target stimulus (e.g., angry face). By contrast, they were instructed to withhold pressing a button in the no-go trials (e.g., fearful faces). The go and no-go trials comprised 50% of the task. In the non-emotional block, the participants were required to identify the sex of the neutral face picture, and the block consisted of two types of non-emotional tasks (go-man and no-go-woman, or go-woman, and no-go-man). We recorded their accuracy rates and reaction times in the emotional and non-emotional go/no-go tasks. The control block comprised a sensorimotor go/no-go task with similar instructions, in which the participants responded to geometric shapes (square or circle). We assessed the task performance using the false alarm error rate (the number of incorrect response/all correct withholding to no-go trials), omission error rate (the number of incorrect no response/all correct responses to go trials), and reaction time (for correct hits) for each block condition. For different performance parameters, reaction time and false alarm indicate impulsivity while omission error rate indicates maintenance of attention.

We measured the participants’ performance in a VFT, which consisted of a 30-s pre-task baseline period, 60-s word production period comprising three 20-s blocks, and 70-s post-task baseline period (33). During the baseline period, we instructed the participants to repeatedly vocalize the five Japanese vowels sequentially. During the word production period, we instructed them to generate the maximum words possible for a particular Japanese mora (rhythmic phonetic unit in the Japanese language). The words were recorded on a digital recorder, and repeats as well as words inflected for the tense or number based on an earlier word were excluded while calculating the total number of words as the measure of the task performance.

We used a computer-based N-back task to measure their working memory performance, as described in our previous study (36). In this task, the participants were displayed a sequence of visual stimuli (random shapes) and had to judge if the current stimulus was identical to the one presented in n positions in the sequence. The shapes were displayed in black and centrally presented on a gray background for 500-ms each, followed by a 2, 500-ms interstimulus interval. We instructed them to rapidly press a predefined key for the targets, and no response was required for the non-targets. The percentages of correct responses and response times (ms) were used for the data analysis.

**FIGURE 4:**
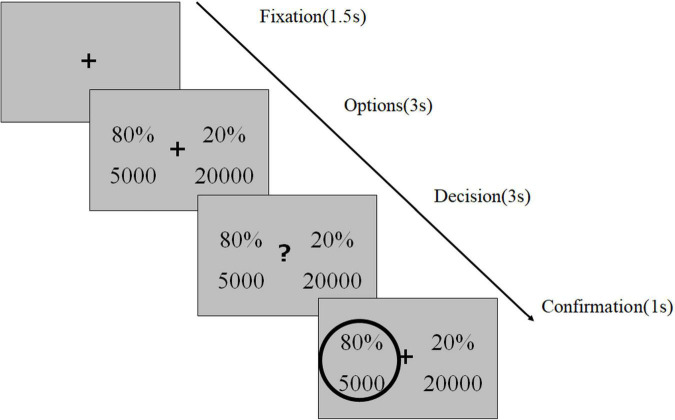
Experimental design of the risk-based decision-making task.

To identify the risk preferences of the patients with AUD, we used a computer-based risk-based decision-making task (Figure 4), as described in our previous study (17). This task consisted of 120 trials conducted in three sessions, each separated by a short break. In each trial, the participants were instructed to select between two gambling options to maximize their rewards. Each option consisted of a reward magnitude (in JPY, the lower number) and the probability of receiving the magnitude of the reward (the upper number). To ensure that the participants were focusing on the task, we inserted a test trial (a total of eight) with a correct answer (e.g., 30%, 5,000 vs. 50%, 5,000) after a randomly selected trial every 15 trials. Following a 1.5-s fixation phase (or inter-trial interval), the stimuli were displayed on the screen for 3 s (option phase), following which a question mark appeared and the participants rapidly indicated their choice by pressing one of the two arrow keys within 3 s and decided which option to select (decision phase). The selected option was highlighted by a gray frame (confirmation phase). We informed the participants that failure to respond within the decision phase would be considered as having no response, and would lead to no reward in the trial. We performed computational modeling to simulate the risk-based decision-making process, in which we estimated a utility function parameter λ and probability weighting parameter (i.e., the one-parameter Prelec weighting function). The utility function parameter (λ) of 1, < 1, and > 1 represented risk-neutrality, risk-aversion, and risk-seeking, respectively. For the probability weighting function parameter (γ), 1, < 1, and > 1 indicated rational probability weighting, the overweighting of small probabilities and underweighting of large probabilities, and the opposite, respectively. The most common maximum likelihood estimation method was used for the model fitting.

### Statistical analyses

We used the geometric shapes task as a control task in addition to the target task (emotional or non-emotional task) in fNIRS. To measure the effect of emotional stimuli on specific brain responses, we extract the results of brain responses during the neutral face task from that of brain responses during the emotional face task. We compared the demographic data or all outcomes of behaviors or brain activations between the two groups using the Mann–Whitney *U*-test or the Student’s *t*-test. Sex distribution was compared using the chi-squared test. Spearman’s rho method was used to perform a correlation analysis of clinical variables and the integral value of (oxy-Hb) in the three brain areas. We performed a binomial logistic regression analysis following the standardization of variables to predict prognosis 6 months following discharge from the hospital.

All statistical analyses were performed using the R ver. 4, Jamovi ver. 2.0, and MATLAB 2018b. Statistical significance was set at *P* < 0.05.

## Results

### Demographic and clinical variables

We recruited 67 patients with AUD. Of these patients, there were 26 dropouts, including two deaths, eight uncontrollable diseases, and 16 who were unable to perform the test.

Forty-one participants (men = 35, 85.4%) completed the 6-month follow-up and were included in the analysis. Of them, 24 (58.6%, men = 21) were sober, and 17 (41.4%, men = 14) relapsed within the first half year of discharge. Table 1 summarizes the characteristics of the abstainer and relapse groups. There were no significant differences (all *p*s > 0.05) between the groups in the background factors and psychiatric symptom rating scales. Ten out of 41 patients with AUD had comorbid illnesses. Of the 24 abstainers, eight had comorbid psychiatric disorders (5 with major depressive disorder, two with dysthymia, and one with major depressive disorder and anxiety disorder). Two relapsers had major depressive disorder. Of the 41 patients, 10 (24.4%) patients each were under antidepressants and sleeping pills, and seven (17.1%) patients each were under antipsychotics and anxiolytics.

**TABLE 1:** Sociodemographic data of the participants.

	Abstainers (*n* = 24)	Relapsers (*n* = 17)	*P*-value
Age	55.0 ± 8.7	51.6 ± 9.3	0.24
Sex (male/female)	21/3	14/3	0.49
Age at first drinking (years)	20.0 (18.0–20.3)	18.0 (16.0–20.0)	0.077
The age of onset (years)	47 (34.8–56.5)	40.0 (35.0–50.0)	0.33
The duration of illness (years)	7.5 (2.8–13.3)	6.0 (3.0–15.0)	0.92
The number of drinking days (per week)	7.0 (6.0–7.0)	7.0 (5.0–7.0)	0.91
The daily intake of alcohol (g)	103.8 ± 34.9	140.0 ± 74.0	0.074
HAND	100.0 (80.0–100.0)	100.0 (80.0–100.0)	0.76
The duration of education (years)	12.0 (12.0–14.3)	12.0 (12.0–14.0)	0.56
SIGH-D 17	3.5 (2.0–6.0)	6.0 (1.0–8.0)	0.55
BDI-II	11.5 (9.0–9.3)	12 (7.0–18.0)	0.68
ARRS-SV	14.8 ± 4.5	14.9 ± 4.1	0.92
BIS-11	62.0 ± 9.8	63.6 ± 14.0	0.67
AUDIT	21.3 ± 7.0	23.2 ± 6.8	0.39
Comorbid illness	8	2	0.11

Date represent median [inter-quartile range (25–75%)] or mean ± standard deviation. HAND: Edinburgh handedness inventory. SIGH-D 17, structured interview guide for the Hamilton depression rating scale-17; BDI-II, beck depression inventory-II; ARRS-SV, alcohol relapse risk scale-stimulus-induced vulnerability; BIS-11, Barratt impulsiveness scale 11th; AUDIT, alcohol use disorders identification test.

### fNIRS

**FIGURE 5:**
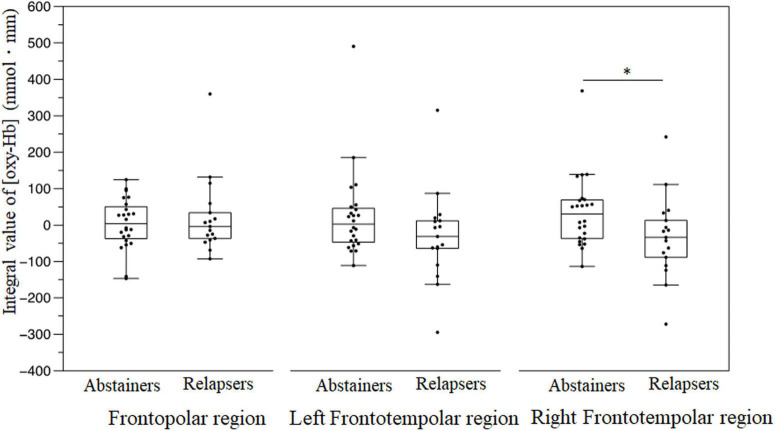
Brain activation displayed as the integral value of (oxy–Hb) in the frontopolar, left frontotemporal, and right frontotemporal regions during the emotional go/no-go task. *P < 0.05.

Compared with the abstinent group, the patients with relapse displayed significantly decreased (oxy-Hb) changes in the right frontotemporal region during the emotional go/no-go task [relapse group: −33.6 (−88.4 to 12.7) vs. abstinent group: 30.5 (−35.8 to 68.3), *U* = 121, *p* = 0.028] (Figure 5). In contrast, there were no significant (oxy-Hb) changes during the VFT between the groups in three regions (all *p* > 0.05).

In the behavior performance, the reaction time to non-emotional stimuli in patients with a relapse was significantly shorter than that in the abstinent group (*U* = 96.0, *p* = 0.004) (Table 2). There was no significant difference in the reaction time to emotional stimuli between the groups. The VFT results did not reveal a significant difference in the number of words generated between the groups (*p* = 0.473) (Table 2).

**TABLE 2:** Behavioral data of the participants.

	Abstainers (*n* = 24)	Relapsers (*n* = 17)	*P*-value
False alarm error (%) (emotional go/no-go task)	3.13 (0.00–9.38)	3.13 (0.00–9.38)	0.848
Omission error rate (%) (emotional go/no-go task)	6.25 (2.34–10.16)	3.13 (3.13–9.38)	0.627
Reaction time (ms) (emotional stimulus)	724.81 (673.99–794.98)	659.81 (621.25–775.79)	0.186
Reaction time (ms) (non-emotional stimulus)	600.29 (555.06–656.26)	528.73 (468.98–568.33)	0.004*
Generated words (verbal fluency task)	14.5 (9.75–17.3)	14 (9.00–16.0)	0.473

*P < 0.05. Date represent the median [inter-quartile range (25–75%)].

### Computer-based cognitive tasks

In the 1- and 2-back trials, there were no significant differences between the groups, with respect to the reaction time and the percentage of correct responses (all *p* > 0.05).

**FIGURE 6:**
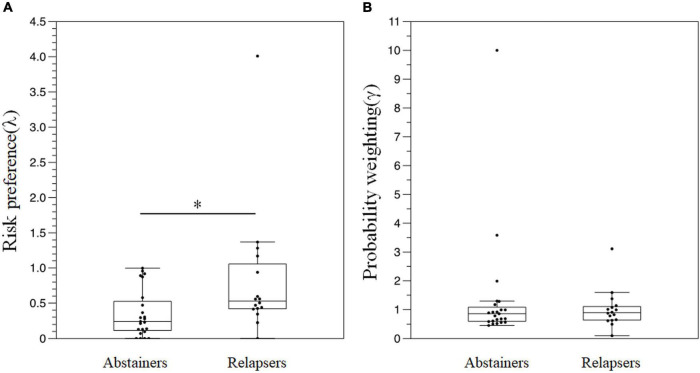
A comparison of the results in the risk-based decision-making task between the groups. **(A)** λ **(B)** γ. *P < 0.05.

Figure 6 depicts the results of the risk-based decision-making task. The relapse group displayed significantly higher λ than the abstainer group [relapser: 0.530 (0.422–0.996) vs. abstainer: 0.241 (0.114 to 0.525), *p* < 0.01] (Figure 6A). There were no significant differences in γ between the groups (all *p* > 0.05) (Figure 6B).

### Associations between brain activation and clinical variables

**FIGURE 7:**
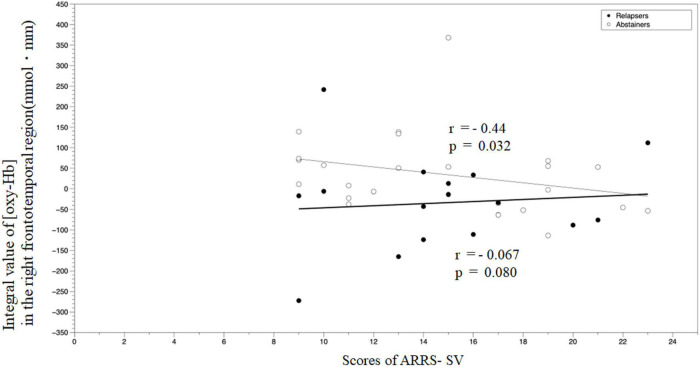
A correlation analysis of the integral value of (oxy–Hb) in the right frontotemporal region during the emotional go/no-go task and ARRS-SV. ARRS-SV, alcohol relapse risk scale-stimulus-induced vulnerability.

We examined the correlations between (oxy-Hb) in the right frontotemporal region during the emotional go/no-go task and clinical measures in each group. There were no significant correlations between (oxy-Hb) and the age of onset, the daily intake of alcohol, BDI-II, BIS-11, or AUDIT in the relapse group (ρ = 0.38, *p* = 0.13; ρ = −0.12, *p* = 0.65; ρ = −0.24, *p* = 0.36; ρ = 0.18, *p* = 0.50; and ρ = −0027, *p* = 0.92, respectively) or the abstinent group (ρ = −024, *p* = 0.25; ρ = −0.28, *p* = 0.18; ρ = −0.18, *p* = 0.41; ρ = 0.23, *p* = 0.29; and ρ = 0.16, *p* = 0.45, respectively). In the abstinent group, we observed a negative correlation between (oxy-Hb) and ARRS-SV (ρ = −0.439, *p* = 0.032); however, there was no correlation between similar measures in the relapse groups (ρ = −0.067, *p* = 0.80) (Figure 7).

### Associated factors for predicting prognosis 6 months following discharge

To predict the prognosis 6 months following discharge, we used the drinking status (relapse or abstainer) as a dependent variable. We used age, the AUDIT scores and the age at AUD onset as the independent variables, which have been previously reported as factors contributing to relapse (23, 37). In addition, (oxy-Hb) changes in the right frontotemporal region by fNIRS and λ, which displayed significant differences between the groups, were added as independent variables. We tested linearity in the logit using the Box-Tidwell transformation in SPSS before performing the logistic regression analysis. The interaction terms of all covariate showed linearity (all *p*s > 0.05). Table 3 presents the results of the regression analysis. The sensitivity and specificity were 68.8 and 87.0%, respectively. Lesser the (oxy-Hb) change in the right frontotemporal region, greater the gambling thoughts were likely to relapse (odds ratio = 0.161; odds ratio = 7.037, respectively).

**TABLE 3:** A logistic analysis of the factors associated with a relapse.

Presense of relapse			
	OR	95% CI	*P*-value
(oxy-Hb) change in the right frontotemporal region	0.161	0.038–0.685	0.013*
λ	7.037	1.17–42.42	0.033*
Age of onset	0.918	0.313–2.70	0.877
AUDIT	1.072	0.436–2.635	0.880
ARRS-SV	1.010	0.410–2.489	0.982
Age	0.669	0.222–2.014	0.474

*P < 0.05. AUDIT, alcohol use disorders identification test; ARRS-SV, alcohol relapse risk scale; stimulus-induced vulnerability; OR, odds ratio; CI, confidence interval.

## Discussion

This longitudinal study examined the factors predicting the prognosis of patients with AUD, 6 months following their discharge from the hospital. Patients with a relapse displayed significantly decreased activation in the right frontotemporal region during the emotional go/no-go task, significantly shorter reaction times to non-emotional stimuli, and greater risk preference in the risk-based decision-making task, compared with the abstinent group. Moreover, we observed a negative correlation between (oxy-Hb) and the craving scales. The logistic regression analysis revealed that the risk of relapse increased with smaller (oxy-Hb) in the right frontotemporal region. There were no significant differences in brain activation during the VFT and N-back tasks between the groups.

In the present study, the relapse group demonstrated significantly decreased (oxy-Hb) changes in the right frontotemporal region, compared with the abstinent group. Several previous studies have demonstrated an association between brain response to emotional stimulation and prognosis prediction in AUD. Brislin et al. observed a negative correlation between activation and subsequent alcohol consumption in the left inferior frontal cortex, which was less activated by negative emotional word stimuli than neutral word stimuli in controls (38). Cservenka et al. mentioned that during positive facial expression stimuli, first-degree relatives of patients with AUD displayed reduced activation in the left superior temporal cortex, compared with non-first-degree relatives (39). In a cross-sectional study using the stop signal task, which examined the response inhibition of the frontal lobe as well as the go/no-go task, heavy drinkers displayed significantly lower activation in the right superior frontal cortex than moderate to lower drinkers (40). In other words, the relapse group could display decreased frontal activation to emotional stimuli which may be associated with increased alcohol use. The prefrontal cortex is responsible for various functions, including emotional regulation (41–44). Neural substrates, including emotional dysregulation, are considered the basis for the onset or susceptibility to relapse in AUD (45). In addition, we observed a negative correlation between brain activity and ARRS-SV in the abstinent group, but not in the relapse group, thereby suggesting craving may not activate brain activity in the right frontotemporal cortex in the relapse group. Craving is a core concept in AUD, and refers to strong demand for drinking. An fMRI study reported that the right dorsolateral prefrontal cortex is one of the regions activated in outpatients with AUD during craving regulation tasks (46), consistent with our findings, thus suggesting a correlation between craving and prefrontal cortex activity. Therefore, our findings suggested that right frontal region dysfunction may be a part of the pathophysiology of relapse. A recent meta-analysis of fMRI showed that patients with AUD demonstrated hyperactivation to alcohol cues compared to controls in prefrontal cortex (16). Interestingly, they showed reduced activity in prefrontal cortex after AUD treatment, suggesting that prefrontal activation may be related to craving suppression. Our results also support that prefrontal activity associated with craving suppression may be an indicator of relapse of AUD. Other biological candidates for relapse in AUD include increased impulsivity due to frontal dysfunction. The relapse group displayed significantly shorter reaction time in the non-emotional go/no-go task than the abstinent group in this study. Impulsivity may represent as a result of impaired inhibitory control. Rupp et al. reported that poor response inhibition in the go/no-go task could be a risk factor for early prognostic detection in AUD (4), consistent with our findings. Therefore, the lack of response inhibition may be one of the characteristics of the prospective relapse group. A recent study showed that GMV reductions in the prefrontal cortex were associated with scores of AUDIT (harmful drinking) and increased impulsivity (47). These findings suggest that frontal dysfunction may be one of the mechanisms of recurrence in AUD.

The parameter λ in the risk-based decision-making task was significantly higher in the relapse group than that in the abstinent group. The result suggested that the relapse group was less averse to risks or in other words, more risk-seeking compared to the abstainer group. Risk-based decision-making encompasses the selection of an action from a set of available alternatives (48), and consists of an aspect of executive function related to the ability to adjust the perception of reward and punishment to make a favorable choice (49). The prefrontal cortex reportedly plays an important role in risk-based decision-making (50), and the impairment of risk-based decision-making is considered one of the characteristics of AUD (51). However, the Iowa Gambling Task, a cognitive task commonly used to explore risk-based decision-making deficits, includes multiple risk-based decision-making components, such as reinforcement learning, the loss of aversion, and risk preference. The risk-based decision-making task developed in our previous study was based on a combination of a utility function and probability-weighted function to capture only risk preferences (17). In this study, the task suggested that the abstainers were more averse to risks.

In the present study, there were no significant differences in behavioral and (oxy-Hb) changes during the VFT, which reflected the executive function between the groups. There are a few studies measuring brain function during VFT in patients with AUD. Patients with AUD after detoxification displayed an identical degree of behavioral performance and (oxy-Hb) changes in the frontotemporal region during the VFT as controls (52). In a cross-sectional study, there was no difference in (oxy-Hb) changes in the frontal activity between the pre- and post-detoxified patients with AUD during the VFT, while brain activity was significantly lower in the pre-detoxified patients with AUD compared to controls (53). These findings indicate the possibility of recovery of brain function in patients with AUD after acute intoxication.

In this study, there was no significant difference between the groups in the N-back task, thereby indicating both groups had comparable working memory capacity. Working memory refers the ability to process information in response to external stimuli, which is necessary for several cognitive abilities, such as reasoning, language comprehension, planning, and spatial processing (54). In a 7-month longitudinal study, Cha. did not observe differences in the results of the N-back task between the relapse and abstinent groups, consistent with our findings (55). A meta-analysis demonstrated that patients with AUD who had been abstainers for <1 year displayed cognitive impairment, including working memory, compared with controls (7), suggesting that cognitive function in patients within up to 1 year of abstinence may be as impaired as that of relapsers.

Our study had some limitations. First, the sample size was small. Second, this study utilized fNIRS, in which the measurement area was limited to 3 mm from the brain’s surface. We could not assess the basal ganglia involved in the reward system, which is associated in the pathology of AUD. Third, it was impossible to determine if our findings were attributed to the original traits of patients with AUD or to the effects of alcohol consumption on the brain. Adolescent rats display higher risk preference 3 months following the consumption of a large dose of alcohol (56). Forth, in the emotional go/no-go task, there was no difference in behavioral performance to emotional stimuli in the two groups despite difference in brain response to emotional stimuli. Fifth, about a quarter of the participants in this study had psychiatric comorbidities, which may influence the results. Sixth, we did not have a control group in this study, and we did not know whether the blood flow changes by using fNIRS in the two groups were similar to the control group or not. Seventh, with only 32 trials per run in the emotional go/no-go task, the signal to noise was very low. Despite these limitations, the strength of this study was that it was a longitudinal study that identified the prognostic factors based on the neural basis or pathophysiology of AUD.

In summary, decreased activation in the right frontotemporal region in response to negative emotional stimuli and risk preference in AUD could predict the relapse of AUD 6 months following discharge.

## Data availability statement

The original contributions presented in this study are included in the article/supplementary material, further inquiries can be directed to the corresponding author.

## Ethics statement

The studies involving human participants were reviewed and approved by the Institutional Review Boards of Koryo Hospital and Yamaguchi University Hospital. The patients/participants provided their written informed consent to participate in this study.

## Author contributions

JS, TM, CC, TH, and SN: conceptualization. TM, CC, and SN: funding acquisition. JS, TM, CC, KE, YoF, YuF, MN, KN, and SN: methodology. JS, TM, YoF, YuF, MN, and KN: investigation. JS, TM, CC, and KE: data analysis. TH and SN: supervision. JS: writing – original draft. TM, CC, TH, and SN: writing – review and editing. All authors contributed to the article and approved the submitted version.
